# Towards a Better Characterisation of Leukemic Cells in Chronic Lymphocytic Leukaemia: Cell-Size Heterogeneity Reflects Their Activation Status and Migratory Abilities

**DOI:** 10.3390/cancers13194922

**Published:** 2021-09-30

**Authors:** Gayane Manukyan, Zuzana Mikulkova, Peter Turcsanyi, Jakub Savara, Markéta Trajerová, Zuzana Kubova, Tomas Papajik, Eva Kriegova

**Affiliations:** 1 Department of Immunology, Faculty of Medicine and Dentistry, Palacký University and University Hospital, 77900 Olomouc, Czech Republic; gaya.manukyan@gmail.com (G.M.); zuzana.mikulkova@fnol.cz (Z.M.); jakub.savara@centrum.cz (J.S.); Marketa.Trajerova@seznam.cz (M.T.); 2 Laboratory of Molecular and Cellular Immunology, Institute of Molecular Biology NAS RA, Yerevan 0014, Armenia; 3 Department of Hematology-Oncology, Faculty of Medicine and Dentistry, Palacký University and University Hospital, 77900 Olomouc, Czech Republic; peter.turcsanyi@fnol.cz (P.T.); zuzana.kubova@fnol.cz (Z.K.); tomas.papajik@fnol.cz (T.P.); 4 Department of Computer Science, Faculty of Electrical Engineering and Computer Science, VSB-Technical University of Ostrava, 70800 Ostrava, Czech Republic

**Keywords:** chronic lymphocytic leukaemia, cell-size heterogeneity, pool of leukemic cells, polarisation, migration

## Abstract

**Simple Summary:**

Chronic lymphocytic leukaemia (CLL) is a heterogeneous chronic disease characterised by the clonal expansion of mature CD19^+^CD23^+^CD5^+^ B-cells in blood, bone marrow and lymphoid tissue. Despite the CLL tumour cell population showing considerable heterogeneity in cell size, the functional characteristics of leukemic cells that differ in size have not been explored. The results of our study demonstrate that differences in CLL cell size reflect their activation state, polarisation and migratory capacity, with large CLL cells being more activated, polarised and motile than the small CLL cells present in the CLL cell pool. Our data provide evidence of the importance of cell-size heterogeneity within the CLL cell pool and the dynamics of cell-size changes for disease pathogenesis.

**Abstract:**

Chronic lymphocytic leukaemia (CLL) is a genetically, morphologically and phenotypically heterogeneous chronic disease with clinical variability between patients. Whether the significant heterogeneity of cell size within the CLL population contributes to the heterogeneous features of this disease has not been investigated. The present study aimed to characterise the phenotypic and functional properties of two subpopulations of typical CLL cells that differ in cell size: small (s-CLL) and large (l-CLL) CLL cells delineated by forward scatter cytometry. The s-CLL cells were characterised by the CD5^low^CXCR4^hi^ phenotype, while the l-CLL cells were characterised by the CD5^hi^CXCR4^dim^ phenotype and indicated a higher expression of CXCR3, CD20, CD38 and HLA-DR. The l-CLL cells displayed higher migration activity towards CXCL12, a tendency towards a higher proliferation rate and an increased capacity to produce IgM in the presence of CpG compared with s-CLL cells. When stimulated with CpG and CXCL12, l-CLL cells were characterised by a higher polarisation phenotype and motility than s-CLL cells. Our study revealed that the differences in CLL cell size reflected their activation status, polarisation and migratory abilities. Our data provide evidence of the importance of cell-size heterogeneity within a CLL pool and the dynamics of cell-size changes for disease pathogenesis, thus deserving further investigation.

## 1. Introduction

Chronic lymphocytic leukaemia (CLL) is characterised by the clonal expansion of mature CD19^+^CD23^+^CD5^+^ B-cells in the blood, bone marrow and lymphoid tissue [1]. CLL cells are characterised by their phenotypic, clonal and functional heterogeneity and contain a pool of resting and proliferating CLL subpopulation cells [2,3,4,5,6]. The inverted expression of CD5 and CXCR4 has been indicated as useful for identifying enriched fractions among recently born/divided and elderly/quiescent CLL cells [2,3,7]. This means that the fraction of proliferating, recently born/divided migrating CLL cells has been shown to preferentially express high levels of CD5, while low levels of CD5 are associated with resting, elderly/quiescent circulating cells [3,8]. The overlapping B-cell receptor (BCR) repertoires between CD5^high^ and CD5^low^ cells suggest a dynamic relationship between these two B-CLL cell subpopulations [4]. Studies have shown that CXCR4^dim^CD5^bright^ ‘proliferative’ cells overexpress more ‘cell division’ genes, while CXCR4^bright^CD5^dim^ ‘resting’ cells express higher levels of ‘antiproliferative’ genes, suggesting that the latter subset may represent a distinct self-renewing type from which all clonal members are derived [3,8].

In addition to immunophenotypic heterogeneity, CLL cells differ in size and morphology. Typical CLL cells are mature small and medium-sized lymphocytes with clumped chromatin surrounded by a thin ring of cytoplasm [9]. A small number of studies that have analysed the clinical significance of atypical CLL morphology have revealed the adverse prognostic significance of increased prolymphocyte numbers and their association with markers of poor prognosis, thus predicting a shorter progression-free survival [9,10]. However, no information is available on the characteristics of typical CLL cells based on their size. This study aimed to analyse the biology of small (s-CLL) and large (l-CLL) CLL cells delineated by forward scatter (FSC) flow cytometry, with a primary focus on activation status, immunophenotype, proliferation, polarisation and migration.

## 2. Materials and Methods

### 2.1. Patients and Sampling

Peripheral blood samples were collected from 23 patients with CLL (16 males and seven females; a median age of 65 years). A diagnosis of CLL was established according to the International Workshop on Chronic Lymphocytic Leukaemia guidelines [11]. Of the enrolled patients, eight were treatment-naïve, six had been pre-treated with immunochemotherapy and nine had been treated with novel agents (ibrutinib (IBR), idelalisib). The detailed clinical characteristics of the enrolled patients are shown in Table 1. An additional eight CLL patients were evaluated before the initiation of the IBR treatment and after 8 weeks of therapy.

### 2.2. Cell-Size Evaluation of CLL Cells

The CLL cells of the patients were evaluated by peripheral blood smears after staining using the May–Grünwald–Giemsa protocol. The smears were evaluated with cellSens Entry (Olympus Life Science) imaging software and the CellaVision DM96 (Sysmex Corporation, Kobe, Japan) automated image analysis system at magnifications of 600× and 1000×, respectively.

### 2.3. Surface Expression of Markers on Small and Large CLL Cells

The CLL cells from all the blood samples were stained with optimal concentrations of antibody combinations and directed against the following surface antigens: CD183(CXCR3)-FITC, CD20-PE, CD5-PerCP-Cy5.5, CD38-Pe-Cy7, CD49d-APC, CD19-APC-Cy7, CD184 (CXCR4)-BV421 and HLA-DR-BV510 (all procured from BioLegend), as previously reported [5,15]. Isotype-matched antibodies (BioLegend) were used as negative controls. 

The determination of s-CLL and l-CLL cells was conducted using FSC data and a back-gating strategy. The analysis was performed using a BD FACSCanto II (Becton Dickinson) instrument, and data acquisition was performed using BD FACSDiva software (v.8.0.2; Becton Dickinson). Flow cytometry data were analysed using FlowJo v.X0.7 software (Tree Star, Inc., San Carlos, CA, USA). In all the experiments, a minimum of 10,000 events was counted. The results were expressed as a percentage and mean fluorescence intensity (MFI).

### 2.4. Intracellular ZAP-70 Staining

Whole blood cells were labelled with CD3-FITC, CD5-PerCP-Cy5.5 and CD19-APC-Cy7 antibodies. Thereafter, they were treated with fixing and permeabilising reagents (BioLegend) according to the manufacturer’s instructions, and they were then stained with the PE-conjugated ZAP-70 protein (clone 1E7.2, BioLegend). The quantity of ZAP-70-positive CLL cells was estimated using isotype control and residual ZAP-70-positive T-cells (T method). Through the T method, ZAP-70 was positioned close to the left edge of the T-cell cluster in a ZAP-70/CD3 plot, which was applied to calculate the percentage of CLL-positive cells [16].

### 2.5. Cell-Culture Experiments

Peripheral blood mononuclear cells were isolated using density-gradient centrifugation. CLL cells were then further separated by fluorescence-activated cell sorting (FACS) using a FACSAria Fusion cell sorter (BD Biosciences), which is used for culturing, apoptosis, migration, polarisation and proliferation studies. CLL cell sorting resulted in purity > 95% for CD19^+^CD5^+^ cells, as determined by flow cytometry. Sorted and paired s-CLL cells (the smallest 15% of the CLL cells in the CLL pool) and l-CLL cells (the largest 15% of the CLL cells in the CLL pool) were cultured in the absence or presence of CpG (3 µg/mL) and CD40L (400 ng/mL) in a complete RPMI-1640 medium, which was supplemented with 10% heat-inactivated foetal bovine serum, 2 mM of L-glutamine, 100 U/mL of penicillin and 100 μg/mL of streptomycin for 24 and 48 h.

### 2.6. Migration Analysis

Transmigration of the CLL cells was assessed using polycarbonate Transwell inserts with a 5 μm pore size (Corning Costar). Briefly, the s-CLL and l-CLL cells sorted at 1 × 10^6^/mL each were placed in the upper chamber in the RPMI-1640 medium containing 1% bovine serum albumin (BSA). Inserts were placed into the lower chamber containing RPMI-1640 with 1% BSA in the presence or absence of 200 ng/mL of CXCL12 (BioLegend). After 3 h at 37 °C in 5% CO_2_, the cells migrated into the lower chamber and were counted using a BD FACSCanto II instrument. The migration rate was calculated as the ratio of CXCL12-treated to CXCL12-untreated cells that transmigrated through the insert. 

### 2.7. Proliferation Assay

The proliferation rate was measured using a colorimetric cell cytotoxicity assay (Cell Cytotoxicity Assay Kit; Abcam); 0.5 × 10^5^ sorted s-CLL and l-CLL cells were separately cultured with CpG and CD40L in duplicates in a 96-well plate. The ratio of OD_570_ to OD_605_ was used to determine cell viability. 

### 2.8. Assessment of Apoptosis

The apoptotic rate of the cultured s-CLL and l-CLL cells was assessed by analysing the binding of annexin V-FITC and the incorporation of propidium iodide (PI). Annexin V/PI double-negative cells were considered viable cells, and annexin V+/PI-cells were classified as apoptotic cells. 

### 2.9. Fluorescent Microscopy

A 1:1 mixture of sorted s-CLL and l-CLL cells in a liquid suspension was fluorescently stained for microscopy (similar to the protocol for immunofluorescence in the case of suspension cells provided by ThermoFisher). The cells were fixed with 4% paraformaldehyde for 10 min and permeabilised by 0.5% Triton X-100 in phosphate-buffered saline (PBS) for 4 min. Unspecific binding was blocked with 1% BSA in PBS for 30 min. Then, the cells were incubated for 90 min with primary antibodies, i.e., mouse anti-human CD5 (1:300, clone CD5/54/F6, Abcam) and rabbit anti-human CXCR3 (1:300, polyclonal, Sigma-Aldrich). This was followed by incubation with secondary antibodies, i.e., goat pre-absorbed anti-mouse IgG (H&L, 1:800, Cy5, polyclonal, Abcam) and goat anti-rabbit IgG (H&L, 1:800, Alexa Fluor488, polyclonal, Abcam) for 60 min. The CLL cells were washed with PBS and centrifuged following the completion of all of the above steps. Finally, the cell pellet was mounted using Fluoromount-G mounting media with 4′,6-diamidino-2-phenylindole (DAPI; Invitrogen) to label the nuclear DNA. Images were taken at a final magnification of 600× using Gen5 software (v.3.08.01; BioTec Instruments, Winooski, VT, USA) and a BioTec Cytation 5MV (BioTec Instruments, Winooski, VT, USA) cell-imaging multi-mode reader.

### 2.10. Quantification of IFN-γ and IgM in Cell-Culture Supernatants by Enzyme-Linked Immunosorbent Assay

The interferon gamma (IFN-γ) levels were measured in the supernatants of cultured s-CLL and l-CLL cells using the MAX™ Deluxe Set Human IFN-γ enzyme-linked immunosorbent assay (ELISA; BioLegend). The total IgM levels were determined using a human IgM ELISA kit (Mabtech AB, Sweden).

### 2.11. Time-Lapse Video Microscopy

The s-CLL and l-CLL cells were separately treated with (i) CpG (3 µg/mL) and cultured in a climate-controlled chamber (37 °C and 5% CO_2_) for 24 h or (ii) CXCL12 (200 ng/mL) and placed on a heated (37 °C and 5% CO_2_) microscope stage. Cell polarisation and motility were monitored by the time-lapse video microscopy of cells over 60 min and recorded using Gen5 software and the BioTec Cytation 5MV reader at a final magnification of 200×. The time-dependent changes in cell shape and motility were visually evaluated.

### 2.12. Statistical Analysis

The difference between the groups was analysed using the Mann–Whitney test. A Wilcoxon signed-rank test was used to estimate the effect of inducers within the investigated groups, and correlations were assessed using Spearman’s correlation analysis. The results are expressed as mean ± standard error of the mean. All statistical analyses and the unsupervised principal component analysis (PCA) were performed using R software (www.r-project.org, version 4.1.1, accessed on 8 August 2021).

## 3. Results

### 3.1. The Expression of Surface and Intracellular Markers

**Figure 1:**
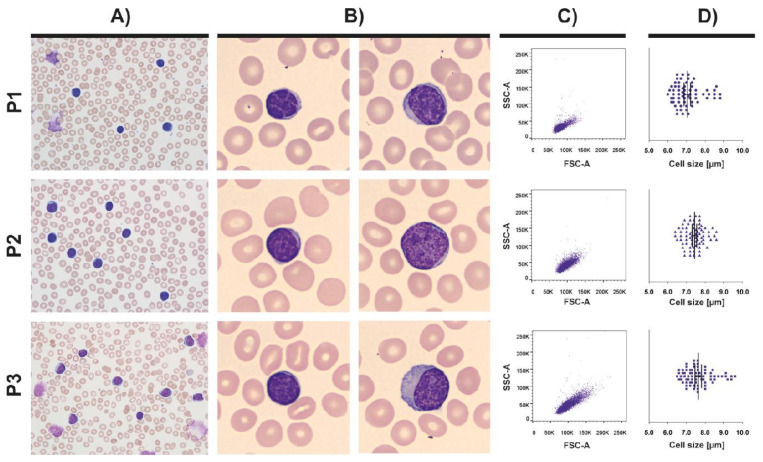
The morphological characteristics of CLL cells. (**A**) Peripheral blood smears from patients with CLL (magnification 600×). (**B**) The s-CLL and l-CLL cells from a pool of typical CLL cells with scant cytoplasm, small round nuclei with condensed chromatin and inconspicuous nucleoli (magnification 1000×). During smear preparation, CLL cells are easily disrupted, creating smudge cells (P1 and P3). (**C**) The distribution of CLL cell sizes within a CLL cell pool based on the forward-scatter (FSC) data using a back-gating strategy. (**D**) The size distribution of CLL cells in a particular patient. Representative examples are shown for three CLL patients (P1–P3).

The size of typical CLL cells ranged from 6.0–9.5 μm; their morphology and size distribution within the CLL cell pool are shown in Figure 1. 

**Figure 2:**
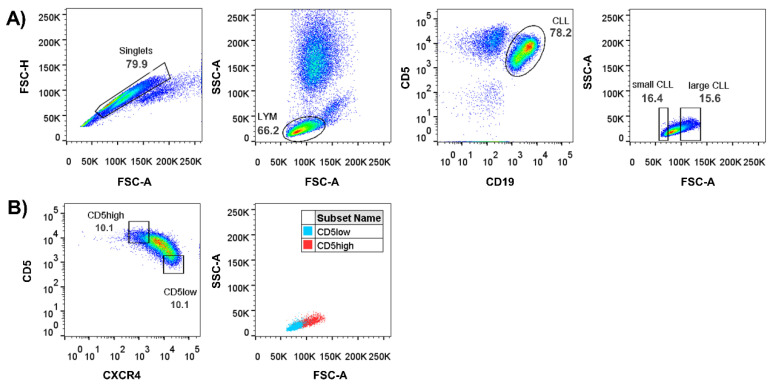
The cytometric analysis for the identification of CLL subpopulations with small and large cell sizes. (**A**) The back-gating strategy for defining s-CLL and l-CLL cells (each constituted ~15% of the CLL cell pool). (**B**) Dot plots showing the positioning of CD5^high^ and CD5^low^ cells (defined by CD5/CXCR4 markers) on the SSC/FSC plot.

In this study, two CLL cell subpopulations with different cell sizes were selected based on the back-gating strategy (Figure 2). Each s-CLL and l-CLL cell population sorted represented ~15% of the CLL cell pool.

**Figure 3:**
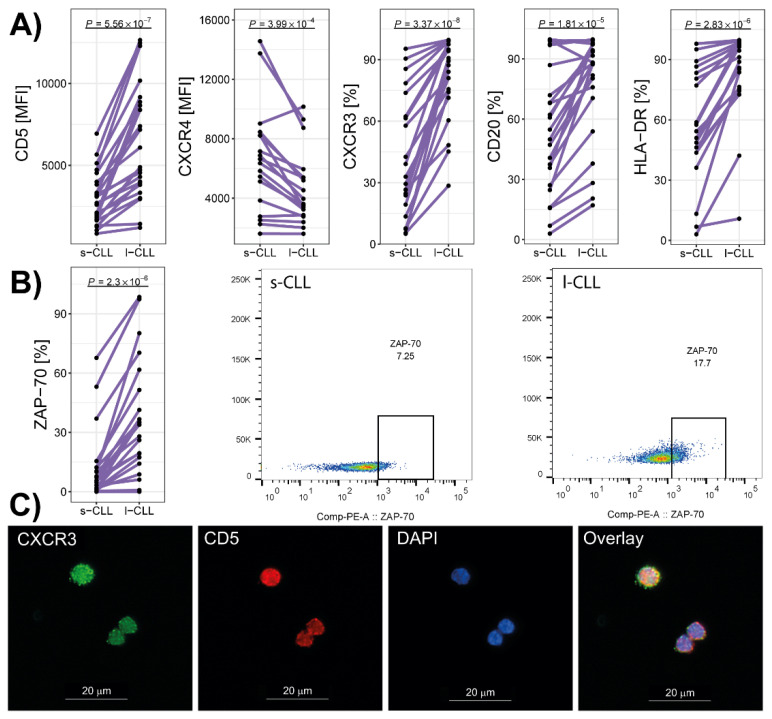
The immunophenotype of s-CLL and l-CLL cells from all the blood samples of the CLL patients. (**A**) The surface expression levels of CD5 and CXCR4, and the percentage of positive cells for CXCR3, CD20, CD38 and HLA-DR. (**B**) The percentage of ZAP-70-positive cells, and representative dot plots of the intracellular expression levels of ZAP-70 in s-CLL and l-CLL cells. (**C**) The double-immunofluorescence staining of s-CLL and l-CLL cells for CXCR3 and CD5; DAPI was used for nuclear staining.

The immunophenotyping of both cell populations revealed that s-CLL cells exhibited low levels of CD5 expression (*p* < 0.001) and high levels of CXCR4 expression (*p* < 0.05) compared with l-CLL cells. Additionally, the percentage of CXCR3^+^, CD20^+^ and HLA-DR^+^ cells was found to be lower in the group of s-CLL cells than in the l-CLL group (Figure 3A). The percentage of CD38^+^ and CD49d^+^ cells did not differ between the studied groups. The intracellular labelling of the cells revealed a lower expression of ZAP-70 in the s-CLL cells than in the l-CLL cells (*p* < 0.01; Figure 3B).

Correlation analyses revealed a significant positive association of CD5^high^ and CD5^low^ cells with the percentages of CXCR3-positive cells, as indicated in our previous study [5]; this was also visualised by immunofluorescent staining (Figure 3C). The percentage of CLL cells in the patients’ blood negatively correlated with the percentage of s-CLL and l-CLL cells that tested positive for HLA-DR (r = −0.59, *p* < 0.01 and r = −0.48, *p* < 0.05, respectively) and CD20 MFI (r = −0.55, *p* < 0.01 and r = −0.56, *p* < 0.01 respectively). In the s-CLL cells, CD5 MFI positively correlated with CXCR3 percentage (r = 0.51, *p* < 0.05); in the l-CLL cells, CD5 MFI positively correlated with both CXCR3 percentage (r = 0.51, *p* < 0.05) and ZAP-70 MFI (r = 0.46, *p* < 0.05). 

**Figure 4:**
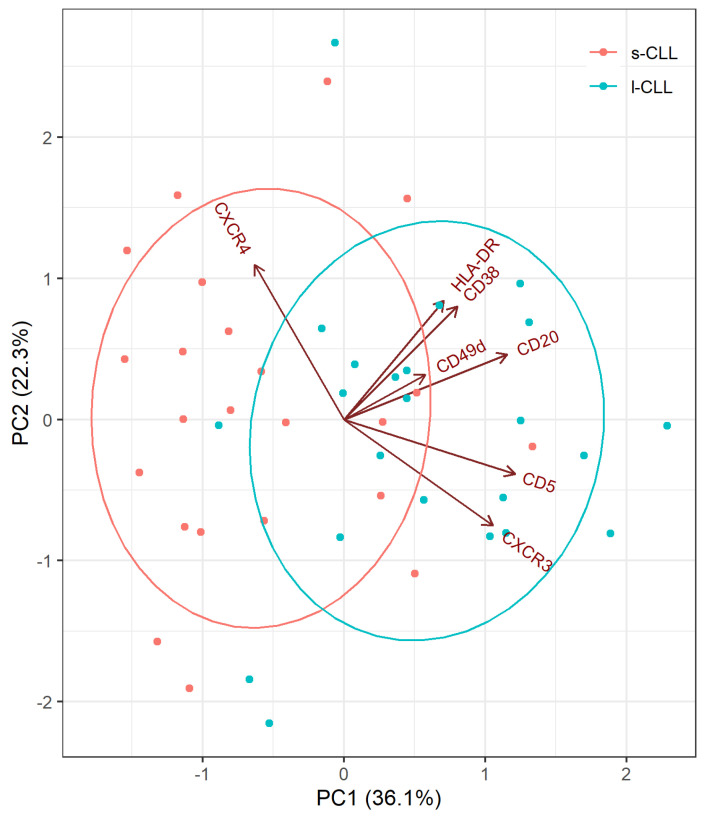
The PCA analysis of surface marker expression in s-CLL and l-CLL cells. Each dot represents a score for the s-CLL (blue) and l-CLL (pink) cell populations derived from the studied CLL patients (*n* = 23). The model loadings are represented by vectors and indicate how each surface marker contributes to the cell variability in a specific direction. The percentage given is the explained variance per principal component of a patient.

To determine if the s-CLL and l-CLL subpopulations were distinct within the expression profiles, PCA was applied. The analysis results revealed the dense clustering of the two subpopulations with few dispersed cases; 66.7% of the cases were correctly classified in the s-CLL and l-CLL subsets. Of the studied markers, the CXCR3, CXCR4, CD20 and CD5 markers had the most consistent ability to correctly classify s-CLL and l-CLL cells (Figure 4). 

### 3.2. The Effects of CpG and CD40L on the Phenotype of Small and Large Chronic Lymphocytic Leukaemia Cells 

**Figure 5:**
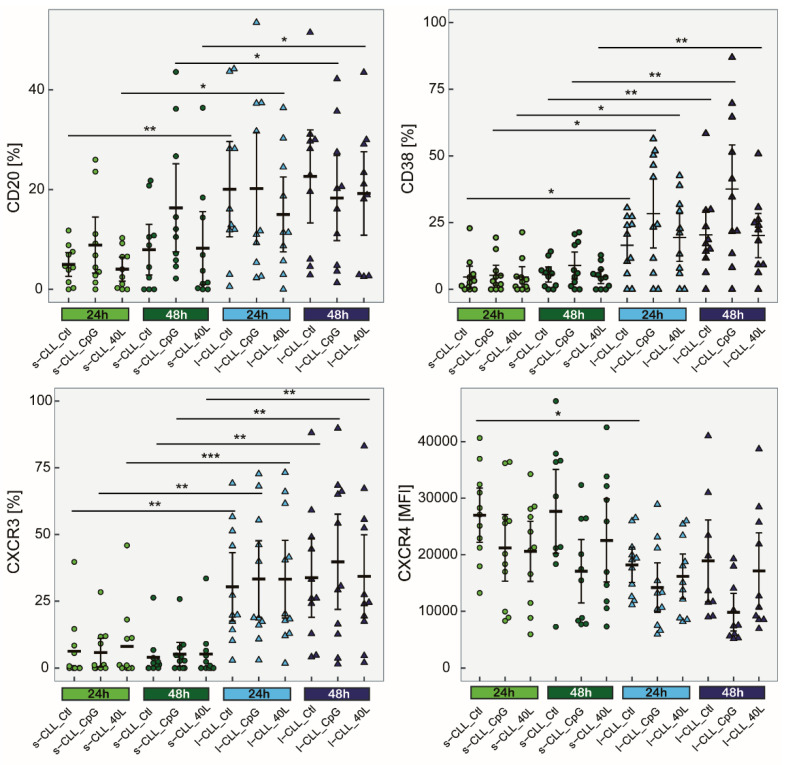
The immunophenotypes of sorted s-CLL vs. l-CLL cells cultured for 24 and 48 h with CpG and CD40L; CXCR4 MFI and the percentage of CXCR3, CD20 and CD38 on cultured CLL cells (*n* = 11). * *p* < 0.05, ** *p* < 0.01, *** *p* < 0.001.

The sensitivity of CLL cells to stimulation may reflect distinctive phenotypic and functional cell subsets. To illustrate the phenotyping features of cultured s-CLL and l-CLL cells, they were left untreated or stimulated with CpG and CD40L. The cultured s-CLL and l-CLL cells retained their expression patterns, which were observed at the basal level and responded to the inducers in a similar manner (Figure 5). 

In particular, an increased percentage of positive l-CLL cells for CXCR3 and CD38 was observed for all stimulated and unstimulated samples compared with s-CLL cells. An increased percentage of CD20 in l-CLL cells was observed only when the cells were left untreated or when they were treated with CD40L at both 24 and 48 h of cultivation. The differences in CXCR4 expression between s-CLL and l-CLL cells were less significant. In contrast to other markers, l-CLL cells, which were left untreated for 24 h, exhibited a decreased expression of CXCR4 (*p* < 0.05) compared with s-CLL cells. 

### 3.3. The Migration Rate of Small and Large Chronic Lymphocytic Leukaemia Cells 

**Figure 6:**
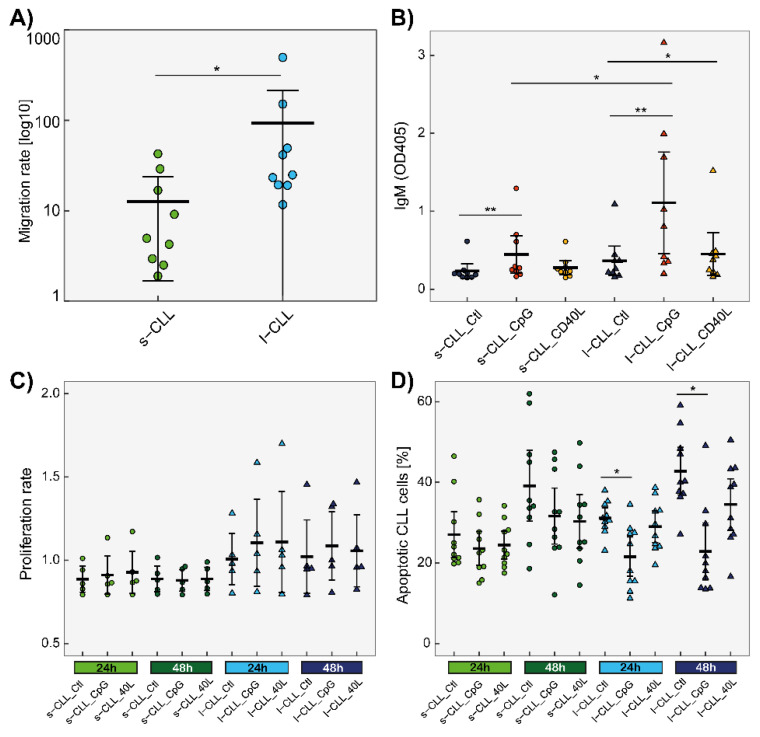
The functional differences between s-CLL and l-CLL cells. (**A**) The migration rate of the sorted s-CLL and l-CLL cells (*n* = 9). The migration rate was calculated as the ratio of CXCL12-treated cells to untreated cells that transmigrated through the insert. y-axis is displayed in logarithmic scale. (**B**) The IgM production in the supernatants of cultured s-CLL and l-CLL cells (*n* = 8); 1 × 10^6^ sorted cells were cultured in the absence or presence of CpG or CD40L for 48 h. The OD_405_ values are plotted without subtracting the background, as determined by ELISA. (**C**) The cell viability (proliferation) of cultured CLL cells (*n* = 9); 0.5 × 10^5^ sorted cells (*n* = 5) were cultured in the absence or presence of CpG and CD40L for 24 and 48 h. The proliferation rate was calculated as the ratio of OD_570_ to OD_605_. (**D**) Apoptosis was measured with annexin V and PI (*n* = 10). Annexin V-positive/PI-negative cells were considered apoptotic. * *p* < 0.05, ** *p* < 0.01.

Next, we estimated the migration rate of the sorted s-CLL and l-CLL cells towards CXCL12. We observed that l-CLL cells migrated faster than s-CLL cells in this context (Figure 6A).

### 3.4. Effects of CpG and CD40L on Proliferation and Apoptotic Rates

Next, we analysed the viability of CLL cells of different sizes and the effects of CpG and CD40L in terms of cell survival and apoptosis. There were no differences in either proliferation or apoptotic rates when comparing s-CLL and l-CLL cells (Figure 6C,D). Although the statistical analysis did not reveal differences in the proliferation rate between the studied cell populations, a tendency for increased proliferation was observed in the group of 1-CLL cells, which were also more sensitive to CpG treatment because of their apoptotic rate, which was observed as being lower than the untreated control samples. 

### 3.5. Production of IFN-γ and IgM

The levels of IFN-γ produced by the cultured CLL cells (sorted from seven CLL patients) were in most cases below the limit of detection. Only in seven samples were the levels of IFN-γ above the limit of detection (4 pg/mL). In six of these seven samples, IFN-γ was produced by l-CLL cells that had been treated either with CpG (3) or CD40L (3), and in only one case, IFN-γ was produced by s-CLL cells treated with CD40L.

The IgM levels were detectable in the supernatants, and significant differences in the IgM levels were observed between the s-CLL and l-CLL cells treated with CpG. The highest amount of IgM was measured in the supernatants of the l-CLL cells stimulated with CpG (Figure 6B). An increase in IgM production was also observed between the CpG-treated s-CLL and l-CLL cell populations compared with their untreated counterparts.

### 3.6. Analysis of Chronic Lymphocytic Leukaemia Cell Polarisation 

**Figure 7:**
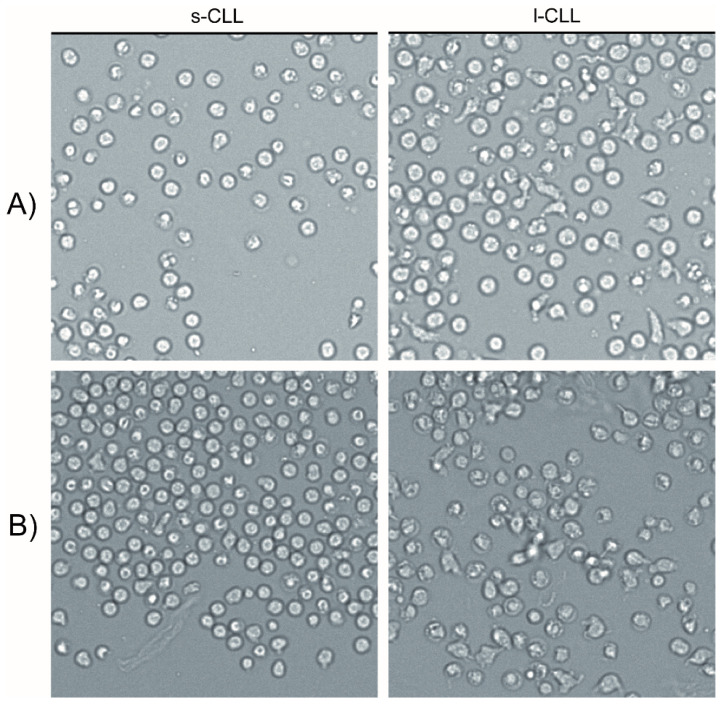
Time-lapse video-microscopy of the sorted s-CLL and l-CLL cells stimulated with (**A**) CpG for 24 h and (**B**) CXCL12 (representative examples). For the time-dependent changes in cell shape and motility see Supplementary Videos S1–S4.

The motility of cells over a 60 min period was determined by the sequential tracing of CLL cell outlines on a video screen for both s-CLL and l-CLL cells stimulated with CpG and CXCL12. An increased proportion of morphologically polarised cells was observed among the l-CLL cells, with a changing cell shape (Figure 7) and increased motility. 

The s-CLL cells were considered to be less motile because of the minimal positional changes of the cells, which had a less deformable cell shape with both the CpG (Supplementary Video S1) and CXCL12 stimulants (Supplementary Video S2). The l-CLL cells were therefore considered to have become more polarised and motile when stimulated with CpG (Supplementary Video S3) and CXCL12 (Supplementary Video S4).

### 3.7. Analysis of CLL Cell Sizes in Treatment-Naïve and Treated CLL Patients 

**Figure 8:**
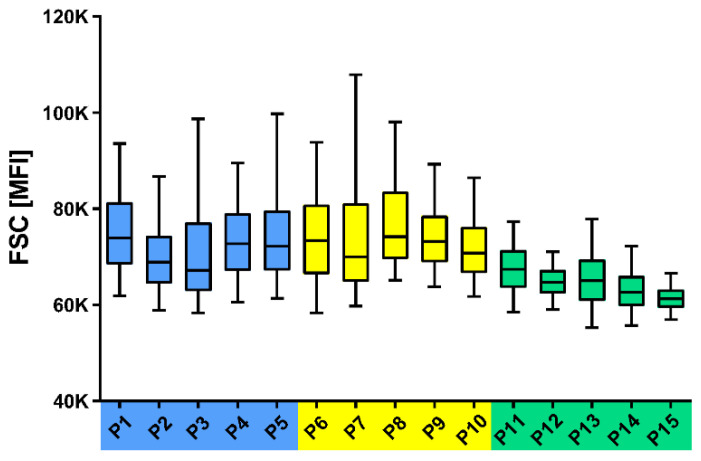
Distribution of CLL cell sizes (expressed as FSC) in the peripheral blood of treatment-naïve CLL patients (P1–P5, blue), patients after immunochemotherapy (P6–P10, yellow) and those receiving ibrutinib (IBR) treatment (P11–P15, green). A representative five patients from each group are shown. The boxplot shows the median, the ends of the boxes indicate the lower (Q1) and upper (Q3) quartiles and the whiskers represent the minimal and maximal values.

Next, we evaluated the size of CLL cells in the peripheral blood of treatment-naïve patients and patients treated with immunochemotherapy and IBR. Analyses showed that treatment-naïve and immunochemotherapy-treated patients had a higher proportion of l-CLL cells than IBR-treated patients, who demonstrated small CLL cell size (expressed as FSC) and narrow CLL cell-size distribution (Figure 8). 

**Figure 9:**
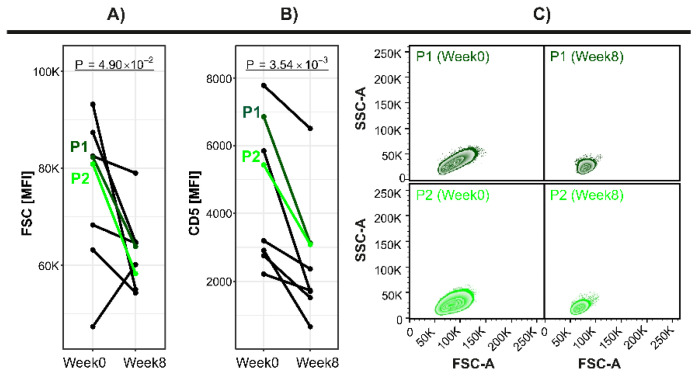
The impact of ibrutinib (IBR) treatment on CLL cell size and phenotype. The patients (*n* = 8) were sampled before the initiation of IBR therapy and after 8 weeks of IBR therapy. (**A**) The distribution of CLL cell sizes (expressed as FSC), (**B**) CD5 expression in individual patients and (**C**) the SSC/FSC dot plots in two representative CLL patients (P1, P2) before and after 8 weeks of IBR treatment.

Moreover, we investigated the impact of IBR treatment on CLL cell size and their phenotype in paired samples before the initiation of IBR therapy and after 8 weeks of IBR therapy. After 8 weeks of IBR, the CLL cell sizes (expressed as FSC) became smaller compared with the samples before the initiation of IBR therapy (*p* < 0.05; Figure 9A). In addition, CD5 expression, which is a marker that is highly correlated with CLL cell size, was reduced after IBR treatment (*p* < 0.01; Figure 9B,C).

## 4. Discussion

Recent findings highlight the importance of heterogeneity in tumour cell size and cell functional plasticity in cancer for influencing the disease phenotype, invasion and progression [17,18]. Whether CLL cell size reflects the biology of neoplastic processes in CLL and contributes to the functional heterogeneity of CLL tumour cells, as well as to the variable clinical manifestations, remains unknown. 

Accordingly, this study focused on the in-depth characterisation of CLL cells that differ in size. The cells were selected from a pool of typical CLL cells in which s-CLL cells were up to a third smaller in size than l-CLL cells. The immunophenotyping demonstrated that s-CLL cells were characterised by the CD5^low^CXCR4^hi^ phenotype, while l-CLL cells were characterised by the CD5^hi^CXCR4^dim^ phenotype and had a higher expression of CXCR3, CD20, CD38 and HLA-DR than s-CLL cells. The migration ability of the s-CLL and l-CLL cells was then investigated, which demonstrated that the latter migrated faster than the former. Our observation in this regard is in line with existing studies [3,8]. In the study conducted by Calissano et al. [3], CD5^hi^CXCR4^dim^ cells expressed higher levels of trafficking-related molecules than CD5^low^CXCR4^hi^ cells. The study conducted by Pasikowska et al. [8] employed a physiologically relevant in vitro circulation system of CLL and reported that neoplastic cells with up-regulated CD5, CD49d, HLA-DR, CD38, CD80, CD86 and CD69 had the highest migratory potential. Our data also showed that after stimulation with CpG oligonucleotides and chemokine CXCL12, l-CLL cells adopted a polarised cell shape and increased their motility in comparison with s-CLL. Whether 1-CLL cells represent a subpopulation of CLL cells with higher migratory capabilities (including their homing abilities), as indicated in vitro and in vivo for morphologically polarised CLL cells [19], deserves further research. 

Interestingly, our in vitro model showed that l-CLL cells with a lower CXCR4 expression had higher migratory capabilities towards CXCL12 than s-CLL cells with the CD5^low^CXCR4^hi^ phenotype. A recent study in vivo using a mouse model demonstrated that only a small fraction of both CD5^hi^CXCR4^dim^ and CD5^low^CXCR4^hi^ CLL cells sorted from peripheral blood was able to infiltrate bone marrow and the liver, but CD5^low^CXCR4^hi^ cells had a higher capacity for homing to immune niches in the spleen [20]. This highlighted the complexity and ambiguity of migration processes in CLL between blood, bone marrow and lymphoid tissue, which are not yet fully understood. It is known that CXCR4 activation can drive cell migration; however, as indicated by our results, CXCR4 expression in l-CLL cells was still high enough for successful migration (at least, in experimental conditions). Second, as proposed, the CD5^hi^CXCR4^dim^ fraction comprised newly born cells and recent arrivals from tissue sites, particularly from lymph nodes [2,3,7]. The reduced expression of CXCR4 in l-CLL cells may have been the result of recent exposure to CXCL12 within tissue or of stimulation. Third, CXCR4 expression by itself is not sufficient for ensuring an effective chemotaxis response to CXCL12. Other molecules may contribute to cell activation, migration to peripheral blood, balancing survival in peripheral blood and the decision to return to the immune niches, thereby influencing B [21] and CLL cells’ [19,20] chemotaxis. As previously proposed [3] and illustrated herein, CD5^hi^ CLL cells were activated, and their expression could be induced in B-cells in vitro [22]. The up-regulation of ZAP-70 and CD38 expression was shown to enhance CLL cell adhesion and the migratory response towards CXCL12 [23,24,25,26]. Similarly, CD20 was shown to be involved in CXCL12-triggered adhesion in the migration of neoplastic B-cells [27]. Furthermore, ZAP-70 was linked to the activation and proliferation of CLL cells [28,29], thus maintaining an ability to be stimulated through the BCR and to enter the cell cycle [30]. Finally, the proportion of migrating cells in our model, as with the study by Pasikowska et al. [8], was very small, indicating the involvement of factors other than CXCR4 in migratory processes, as recently reported by Seda et al. [20]. 

Similar to normal B-cells, CLL cells differ in size. The relative differences in B-cell sizes have been shown to be dependent on age and the stage of maturation [31]. The larger cell size of CD5^hi^CXCR4^dim^ CLL cells was likely caused by the activation status of the cells. The activation of BCR is critical for the survival, proliferation and trafficking of CLL cells [32]. In our study, larger cell size was accompanied by a higher expression of CD5, the hyper-expression of activation markers, higher production of IgM and IFN-γ and cell proliferation and migration, which is consistent with the activated status of the cells. It was shown that increased S6K1 activity and the amplified synthesis of cellular proteins were accompanied by a substantial increase in B-cell size [33]. Similarly, the induction of activation markers, including major histocompatibility complex II molecules on B-cells, was associated with the increase in cell size [34]. It is noted that an increased immature B-cell size was shown to be correlated with CD5 levels in mice during B-cell maturation [35]. Proliferation processes are also associated with an increase in mass and size before initiating cell division. Following our study, the highest proportion of proliferation events was observed in a subset of CD5^hi^CXCR4^low^ subpopulation CLL cells in vivo [7] and in vitro [3,29]. Concurrently, the apoptotic rate of CD5^hi^CXCR4^low^ proliferation cells did not decrease in our experiments; only the TLR9 agonist CpG gave rise to a reduced apoptotic rate of l-CLL cells. Similar to the findings of a recent study [36], CD40L was found to be a weak factor in the prevention of apoptosis. The immunostimulatory potential of CpG and CD40L on both CLL cell subclones was comparable, thus supporting the hypothesis that the molecular heterogeneity of CLL cells of different sizes is a dynamic and interchangeable process that reflects the activation status of the cells and their trafficking history. 

Interestingly, CLL cells from IBR-treated patients, compared with untreated and immunochemotherapy-treated patients, were characterised by smaller sizes. The beneficial effect of IBR on the function of immune and CLL cells themselves is well established [37]. In particular, along with the redistribution of CLL cells from the spleen and lymph nodes into the circulation, IBR inhibits their activation and proliferation [38,39] and impairs CXCR4 signalling [40]. According to our data, this ‘compartment shift’ of CLL cells during IBR treatment is accompanied by a reduction in CLL cell size and CD5 expression, which reflects their activation status. Our data enabled us to hypothesise that IBR already inhibits CLL cell activation in stromal niches, resulting in an enhanced fraction of small CLL cells in the circulation. Our study therefore adds to a growing body of evidence demonstrating that IBR exerts a significant modulatory effect on CLL cells, although the exact mechanism in the various compartments should be further investigated.

This study has several limitations. First, a modest sample size enrolled in the study did not enable us to perform a sub-analysis of the association between cell size and genetic abnormalities. Second, the expression of the studied markers was not corrected according to cell size. However, distinct phenotypes, the functional differences and an inverse expression of CXCR4 in CLL cells of different sizes support the fact that the expression levels of the studied markers are only partly size-dependent and result mainly from differences in the biological states of CLL cells. Third, this study focused only on circulating CLL cells, not bone marrow or lymphoid tissue cells.

## 5. Conclusions

In our study, the immunophenotype differences observed between CLL populations, based on size differences, were corroborated by the marked differences in their biological functions. The results indicate that the functional differences observed in vitro may reflect a different in vivo leukemic potential, which is likely responsible for the heterogeneous features of the disease and may contribute to different clinical manifestations in CLL patients. An in-depth understanding of the heterogeneity of CLL populations may contribute to a better understanding of disease heterogeneity in CLL and the development of novel effective therapeutic approaches to this disease.

## Figures and Tables

**Table 1:** Characteristics of the CLL patients.

Parameters	CLL Patients (*n* = 23)
Age in years: median (min–max)	65 (51–84)
Gender: male/female	16/7
White blood cell count (×10^9^/L): median (min–max)	112.3 (26.7–498.3)
Percentage of lymphocytes in the peripheral blood: median (min–max)	92.8 (75.7–98.8)
Time from diagnosis to sampling (in months): median (min–max)	96 (6–200)
Binet stage: A/B/C	6/5/12
Bulky lymphadenopathy ≥5 cm: yes/no	5/18
Splenomegaly: yes/no	4/19
*IgHV* mutational status: unmutated/mutated	15/8
Genetic characteristics:	
*TP53* disruption (deletion 17p and/or *TP53* mutations): yes/no/n.a.	6/13/4
del(11q22): yes/no/n.a.	7/15/1
del(13q14): yes/no/n.a.	3/18/2
Treatment: yes/no	15/8
Previous immunochemotherapy:	6
Time from the end of treatment to sampling (in months): median (min–max)	22 (4–68)
Current treatment with iBCR (ibrutinib/idelalisib):	9 (8/1)
Time from the start of iBCR treatment to sampling (in months): median (min–max)	3 (1–10)

Legend: n.a. = not available; iBCR = B-cell receptor (BCR) signalling inhibitors. Next-generation sequencing was used for the detection of *TP53* mutations, Sanger sequencing for *IgHV* mutational status and cytogenetics and FISH analysis for deletion 17p and other aberrations, as previously reported [12,13,14].

## Data Availability

The data that support the findings of this study are available upon reasonable request from the corresponding author.

## Supplementary Materials

The following are available online at https://www.mdpi.com/article/10.3390/cancers13194922/s1, Video S1: Small_CLL cells_CpG, Video S2: Small_CLL cells_CXCL12, Video S3: Large_CLL cells_CpG, Video S4: Large_CLL cells_CXCL12.

## References

[1] Kipps T.J., Stevenson F.K., Wu C.J., Croce C.M., Packham G., Wierda W.G., O’Brien S., Gribben J., Rai K. (2017). Chronic lymphocytic leukaemia. Nat. Rev. Dis. Primers.

[2] Calissano C., Damle R.N., Hayes G., Murphy E.J., Hellerstein M.K., Moreno C., Sison C., Kaufman M.S., Kolitz J.E., Allen S.L. (2009). In vivo intraclonal and interclonal kinetic heterogeneity in B-cell chronic lymphocytic leukemia. Blood.

[3] Calissano C., Damle R.N., Marsilio S., Yan X.J., Yancopoulos S., Hayes G., Emson C., Murphy E.J., Hellerstein M.K., Sison C. (2011). Intraclonal complexity in chronic lymphocytic leukemia: Fractions enriched in recently born/divided and older/quiescent cells. Mol. Med..

[4] Bashford-Rogers R.J., Palser A.L., Hodkinson C., Baxter J., Follows G.A., Vassiliou G.S., Kellam P. (2017). Dynamic variation of CD5 surface expression levels within individual chronic lymphocytic leukemia clones. Exp. Hematol..

[5] Manukyan G., Papajik T., Mikulkova Z., Urbanova R., Smotkova Kraiczova V., Savara J., Kudelka M., Turcsanyi P., Kriegova E. (2020). High CXCR3 on Leukemic Cells Distinguishes IgHVmut from IgHVunmut in Chronic Lymphocytic Leukemia: Evidence from CD5high and CD5low Clones. J. Immunol. Res..

[6] Mikulkova Z., Manukyan G., Turcsanyi P., Kudelka M., Urbanova R., Savara J., Ochodkova E., Brychtova Y., Molinsky J., Simkovic M. (2021). Deciphering the complex circulating immune cell microenvironment in chronic lymphocytic leukaemia using patient similarity networks. Sci. Rep..

[7] Herndon T.M., Chen S.S., Saba N.S., Valdez J., Emson C., Gatmaitan M., Tian X., Hughes T.E., Sun C., Arthur D.C. (2017). Direct in vivo evidence for increased proliferation of CLL cells in lymph nodes compared to bone marrow and peripheral blood. Leukemia.

[8] Pasikowska M., Walsby E., Apollonio B., Cuthill K., Phillips E., Coulter E., Longhi M.S., Ma Y., Yallop D., Barber L.D. (2016). Phenotype and immune function of lymph node and peripheral blood CLL cells are linked to transendothelial migration. Blood.

[9] Oscier D., Else M., Matutes E., Morilla R., Strefford J.C., Catovsky D. (2016). The morphology of CLL revisited: The clinical significance of prolymphocytes and correlations with prognostic/molecular markers in the LRF CLL4 trial. Br. J. Haematol..

[10] Melo J.V., Catovsky D., Gregory W.M., Galton D.A. (1987). The relationship between chronic lymphocytic leukaemia and prolymphocytic leukaemia. IV. Analysis of survival and prognostic features. Br. J. Haematol..

[11] Hallek M., Cheson B.D., Catovsky D., Caligaris-Cappio F., Dighiero G., Döhner H., Hillmen P., Keating M.J., Montserrat E., Rai K.R. (2008). International Workshop on Chronic Lymphocytic Leukemia. Guidelines for the diagnosis and treatment of chronic lymphocytic leukemia: A report from the international workshop on chronic lymphocytic leukemia updating the national cancer institute-working group 1996 guidelines. Blood.

[12] Petrackova A., Vasinek M., Sedlarikova L., Dyskova T., Schneiderova P., Novosad T., Papajik T., Kriegova E. (2019). Standardization of Sequencing Coverage Depth in NGS: Recommendation for Detection of Clonal and Subclonal Mutations in Cancer Diagnostics. Front. Oncol..

[13] Kruzova L., Schneiderova P., Holzerova M., Vatolikova M., Divoka M., Turcsanyi P., Urbanova R., Kudelka M., Radvansky M., Kriegova E. (2019). Complex karyotype as a predictor of high-risk chronic lymphocytic leukemia: A single center experience over 12 years. Leuk. Res..

[14] Obr A., Procházka V., Jirkuvová A., Urbánková H., Kriegova E., Schneiderová P., Vatolíková M., Papajík T. (2018). TP53 Mutation and Complex Karyotype Portends a Dismal Prognosis in Patients with Mantle Cell Lymphoma. Clin. Lymphoma Myeloma. Leuk..

[15] Manukyan G., Papajik T., Gajdos P., Mikulkova Z., Urbanova R., Gabcova G., Kudelka M., Turcsányi P., Ryznerova P., Prochazka V. (2017). Neutrophils in chronic lymphocytic leukemia are permanently activated and have functional defects. Oncotarget.

[16] Rossi F.M., Del Principe M.I., Rossi D., Irno Consalvo M., Luciano F., Zucchetto A., Bulian P., Bomben R., Dal Bo M., Fangazio M. (2010). Prognostic impact of ZAP-70 expression in chronic lymphocytic leukemia: Mean fluorescence intensity T/B ratio versus percentage of positive cells. J. Transl. Med..

[17] Kumar S., Saxena N., Sarkar M., Barai A., Sen S. (2021). Combined heterogeneity in cell size and deformability promotes cancer invasiveness. J. Cell Sci..

[18] Lüönd F., Tiede S., Christofori G. (2021). Breast cancer as an example of tumour heterogeneity and tumour cell plasticity during malignant progression. Br. J. Cancer..

[19] Dampmann M., Görgens A., Möllmann M., Murke F., Dührsen U., Giebel B., Dürig J. (2020). CpG stimulation of chronic lymphocytic leukemia cells induces a polarized cell shape and promotes migration in vitro and in vivo. PLoS ONE.

[20] Seda V., Vojackova E., Ondrisova L., Kostalova L., Sharma S., Loja T., Mladonicka Pavlasova G., Zicha D., Kudlickova Peskova M., Krivanek J. (2021). FoxO1-GAB1 axis regulates homing capacity and tonic AKT activity in chronic lymphocytic leukemia. Blood.

[21] Brandes M., Legler D.F., Spoerri B., Schaerli P., Moser B. (2000). Activation-dependent modulation of B lymphocyte migration to chemokines. Int. Immunol..

[22] Gary-Gouy H., Harriague J., Bismuth G., Platzer C., Schmitt C., Dalloul A.H. (2002). Human CD5 promotes B-cell survival through stimulation of autocrine IL-10 production. Blood.

[23] Deaglio S., Vaisitti T., Aydin S., Bergui L., D’Arena G., Bonello L., Omedé P., Scatolini M., Jaksic O., Chiorino G. (2007). CD38 and ZAP-70 are functionally linked and mark CLL cells with high migratory potential. Blood.

[24] Stamatopoulos B., Haibe-Kains B., Equeter C., Meuleman N., Sorée A., De Bruyn C., Hanosset D., Bron D., Martiat P., Lagneaux L. (2009). Gene expression profiling reveals differences in microenvironment interaction between patients with chronic lymphocytic leukemia expressing high versus low ZAP70 mRNA. Haematologica.

[25] Calpe E., Purroy N., Carpio C., Abrisqueta P., Carabia J., Palacio C., Castellví J., Crespo M., Bosch F. (2013). ZAP-70 promotes the infiltration of malignant B-lymphocytes into the bone marrow by enhancing signaling and migration after CXCR4 stimulation. PLoS ONE.

[26] Laufer J.M., Lyck R., Legler D.F. (2018). ZAP70 expression enhances chemokine-driven chronic lymphocytic leukemia cell migration and arrest by valency regulation of integrins. FASEB J..

[27] Kozlova V., Ledererova A., Ladungova A., Peschelova H., Janovska P., Slusarczyk A., Domagala J., Kopcil P., Vakulova V., Oppelt J. (2020). CD20 is dispensable for B-cell receptor signaling but is required for proper actin polymerization, adhesion and migration of malignant B cells. PLoS ONE.

[28] Purroy N., Abrisqueta P., Carabia J., Carpio C., Palacio C., Bosch F., Crespo M. (2015). Co-culture of primary CLL cells with bone marrow mesenchymal cells, CD40 ligand and CpG ODN promotes proliferation of chemoresistant CLL cells phenotypically comparable to those proliferating in vivo. Oncotarget.

[29] Schleiss C., Ilias W., Tahar O., Güler Y., Miguet L., Mayeur-Rousse C., Mauvieux L., Fornecker L.M., Toussaint E., Herbrecht R. (2019). BCR-associated factors driving chronic lymphocytic leukemia cells proliferation ex vivo. Sci. Rep..

[30] Longo P.G., Laurenti L., Gobessi S., Petlickovski A., Pelosi M., Chiusolo P., Sica S., Leone G., Efremov D.G. (2007). The Akt signaling pathway determines the different proliferative capacity of chronic lymphocytic leukemia B-cells from patients with progressive and stable disease. Leukemia.

[31] Lúcio P., Parreira A., van den Beemd M.W., van Lochem E.G., van Wering E.R., Baars E., Porwit-MacDonald A., Bjorklund E., Gaipa G., Biondi A. (1999). Flow cytometric analysis of normal B cell differentiation: A frame of reference for the detection of minimal residual disease in precursor-B-ALL. Leukemia.

[32] Burger J.A., Gribben J.G. (2014). The microenvironment in chronic lymphocytic leukemia (CLL) and other B cell malignancies: Insight into disease biology and new targeted therapies. Semin. Cancer Biol..

[33] Donahue A.C., Fruman D.A. (2003). Proliferation and survival of activated B cells requires sustained antigen receptor engagement and phosphoinositide 3-kinase activation. J. Immunol..

[34] Waters L.R., Ahsan F.M., Wolf D.M., Shirihai O., Teitell M.A. (2018). Initial B Cell Activation Induces Metabolic Reprogramming and Mitochondrial Remodeling. iScience.

[35] Vanhee S., Åkerstrand H., Kristiansen T.A., Datta S., Montano G., Vergani S. (2019). Lin28b controls a neonatal to adult switch in B cell positive selection. Sci. Immunol..

[36] Hamilton E., Pearce L., Morgan L., Robinson S., Ware V., Brennan P., Thomas N.S., Yallop D., Devereux S., Fegan C. (2012). Mimicking the tumour microenvironment: Three different co-culture systems induce a similar phenotype but distinct proliferative signals in primary chronic lymphocytic leukaemia cells. Br. J. Haematol..

[37] Solman I.G., Blum L.K., Burger J.A., Kipps T.J., Dean J.P., James D.F., Mongan A. (2021). Impact of long-term ibrutinib treatment on circulating immune cells in previously untreated chronic lymphocytic leukemia. Leuk. Res..

[38] Cheng S., Ma J., Guo A., Lu P., Leonard J.P., Coleman M., Liu M., Buggy J.J., Furman R.R., Wang Y.L. (2014). BTK inhibition targets in vivo CLL proliferation through its effects on B-cell receptor signaling activity. Leukemia.

[39] Herman S.E., Mustafa R.Z., Gyamfi J.A., Pittaluga S., Chang S., Chang B., Farooqui M., Wiestner A. (2014). Ibrutinib inhibits BCR and NF-κB signaling and reduces tumor proliferation in tissue-resident cells of patients with CLL. Blood.

[40] Chen S.S., Chang B.Y., Chang S., Tong T., Ham S., Sherry B., Burger J.A., Rai K.R., Chiorazzi N. (2016). BTK inhibition results in impaired CXCR4 chemokine receptor surface expression, signaling and function in chronic lymphocytic leukemia. Leukemia.

