# Supplementary material for: Towards a Better Characterisation of Leukemic Cells in Chronic Lymphocytic Leukaemia: Cell-Size Heterogeneity Reflects Their Activation Status and Migratory Abilities
Source: Cancers (Basel). 2021 Sep 30;13(19):4922. doi: 10.3390/cancers13194922 (PMC8508598; doi:10.3390/cancers13194922)
Supplement: Supplementary file 1 [file cancers-13-04922-s001.zip › Supplementary data/Legend to Videos S1-S4.pdf]

Supplementary material

Towards a better characterisation of leukemic cells in chronic lymphocytic leukaemia: cell size heterogeneity reflects their activation status and migratory abilities

Gayane Manukyan, Zuzana Mikulkova, Peter Turcsanyi, Jakub Savara, Marketa Trajerova, Zuzana Kubova, Tomas Papajik, Eva Kriegova

Legend to Videos S1-S4

Video S1: Small_CLL cells_CpG

Time-lapse video-microscopy of sorted small CLL (s-CLL) cells stimulated with CpG for 24 h. Time-dependent changes in cell shape and motility after stimulation were recorded for 60 min. The video shows 49 photos in 75 sec time intervals.

Video S2: Small_CLL cells_CXCL12

Time-lapse video-microscopy of sorted small CLL (s-CLL) cells stimulated with CXCL12. Time-dependent changes in cell shape and motility after stimulation were recorded for 60 min. The video shows 45 photos in 80 sec time intervals.

Video S3: Large_CLL cells_CpG

Time-lapse video-microscopy of sorted large CLL (l-CLL) cells stimulated with CpG for 24 h. Time-dependent changes in cell shape and motility after stimulation were recorded for 60 min. The video shows 49 photos in 75 sec time intervals.

Video S4: Large_CLL cells_CXCL12

Time-lapse video-microscopy of sorted large CLL (l-CLL) cells stimulated with CXCL12. Time-dependent changes in cell shape and motility after stimulation were recorded for 60 min. The video shows 45 photos in 80 sec time intervals.
